# The Black women first initiative: using implementation science to examine bundled interventions to improve care and treatment coordination for Black women with HIV

**DOI:** 10.1186/s12913-023-09446-z

**Published:** 2023-05-26

**Authors:** Serena Rajabiun, Corliss Heath, Angela Wangari Walter, Judith C. Scott, Alicia Downes, Esther Jennings, Howard J. Cabral, Cecilia Flores-Rodriguez, Linda Sprague Martinez

**Affiliations:** 1 University of Massachusetts Lowell, Lowell, USA; 2 Health Resources and Services Administration, Division of Policy and Data, HIV/AIDS Bureau, Rockville, USA; 3 Boston University School of Social Work, Boston, USA; 4 AIDS United, Washington, USA; 5 Boston University School of Public Health, Boston, USA; 6 Boston University School of Social Work, Center for Emerging Infectious Disease Policy and Research and Clinical Translational Science Institute Community Engagement Program, Boston, USA

**Keywords:** Implementation science, HIV, Black women, Health equity

## Abstract

**Background:**

Black cisgender and transgender women are disproportionately affected by the HIV epidemic compared to women of other racial and ethnic identities. Twelve demonstration sites across the United States are adapting, implementing and evaluating a comprehensive bundle of two or more evidence informed interventions to improve health and outcomes and quality of life for Black women with HIV.

**Methods:**

Guided by Greenhalgh’s Conceptual Model of Diffusion of Innovations in Health Service Organizations and Proctor’s model for use of implementation strategies and evaluating implementation, service and client outcomes, this mixed methods study documents outcomes at the client, organization, and system level. Participant eligibility for the bundled interventions includes: individuals who are 18 years or older, identify as Black or African-American, identify as cisgender or transgender female and have a diagnosis of HIV. Qualitative data are collected systematically through a series of annual site visits and a standardized monthly call form to assess the barriers and facilitators to the implementation process and the key determinants impacting the intervention uptake and implementation strategies. Quantitative data collection for the implementation, service and client outcomes is conducted through a pre-post prospective study to examine the impact on Black women’s health and well-being. Implementation outcomes include: the *reach* to Black women with HIV, *adoption* of interventions across the sites and their community; the *fidelity* to the components of the bundled interventions; the *costs* of the intervention; and the *sustainability* of the intervention in the organization and community. Primary service and client outcomes are improved linkage to and retention in HIV care and treatment, increased and sustained viral suppression, improved quality of life and resilience, and stigma reduction.

**Discussion:**

The study protocol presented is specifically designed to advance the evidence for adopting culturally responsive and relevant care into clinic and public health settings to improve the health and well-being for Black women with HIV. In addition the study may advance the implementation science field by furthering what is known about the ways in which bundled interventions can address barriers to care and facilitate the uptake of organizational practices to improve health.

**Supplementary Information:**

The online version contains supplementary material available at 10.1186/s12913-023-09446-z.

## Background

HIV continues to be a serious public health challenge in the United States. The number of new infections remains at approximately 38,000 per year and not all people benefit equally from advances in HIV prevention and treatment [1]. Black cisgender and transgender women are disproportionately affected by the HIV epidemic compared to women of other racial and ethnic identities. New HIV infections among Black women are 13 times higher than among White women and four times higher compared to Latina women [2]. Black women are less likely to be virally suppressed and more likely to die prematurely due to HIV compared to women who identify as White or Latinx [3–5]. The Ryan White HIV/AIDS Program, the payor of last resort for people with HIV, helps to reduce inequities in outcomes by providing access to care and treatment with recent data indicating Black women in aggregate may have similar rates of viral suppression and retention in care compared to their White and Latinx counterparts [6]. Despite these advances, inequities persist for specific groups of Black women. The populations include persons who identify as transgender, those ages 15–34 years and those experiencing unstable housing having poorer rates of retention in care and viral suppression compared to women with other racial and ethnic identities [6].

Multiple challenges exist for Black women in accessing appropriate HIV care and treatment. Social and structural factors including stigma and racism, lack of social support, poor patient-provider relationships and communication, medical mistrust and unmet social needs (childcare, financial stability, transportation) are among the proximal determinants of the inequities in the health care system experienced by Black women with HIV [7, 8]. Underlying these forces is historical trauma resulting from a legacy of oppression from racism, cisgenderism [9] and transphobia with recent studies indicating their negative impact on care and health outcomes [7, 8, 10–15].

Addressing these challenges to HIV care and treatment requires multilevel interventions that center the preferences and lived experiences of Black women with HIV [11, 12, 16]. However, there are limited studies of evidence based culturally relevant interventions for Black women with HIV to improve viral suppression and engagement in care [11]. Emerging strategies include the use of motivational interviewing techniques to support adherence to care and peers as members of the care team to encourage linkage and retention in care [16–18]. Recent studies show Black women with HIV employing resilience-based strategies, such as building supportive communities through children, grandmothers, peers/friends as coping strategies to face the adversities of stigma and discrimination and to promote health behaviors [19–21]. Further research is needed to identify the mechanisms by which these strategies reduce the challenges to care and treatment and improve the health and well-being for Black women with HIV.

The National HIV/AIDS Strategy (2022–2025) specifically prioritizes efforts to reduce disparities and improve HIV health outcomes for Black women [1]. The Strategy calls for implementation research on creative health care delivery approaches implemented by interdisciplinary teams to enhance generalizable knowledge to a variety of service settings to reach Black women. In addition, the Strategy highlights the need for active community engagement to inform program implementors on how to tailor evidence-based strategies to improve care and treatment for Black women.

As part of the response to the National HIV/AIDS Strategy, the Health Resources and Services Administration (HRSA), HIV/AIDS Bureau (HAB), Ryan White HIV/AIDS Program (RWHAP) Part F Special Projects of National Significance (SPNS) and Minority HIV/AIDS Fund (MHAF) designed and funded the initiative: *Improving Care and Treatment Coordination: Focusing on Black Women with HIV* (2020–2024) (Black Women First Initiative). The initiative addresses social health determinants, utilization of comprehensive HIV care and treatment services, support of continuous care engagement, and improving health outcomes for Black women with HIV in a culturally responsive manner. Twelve demonstration sites were selected through a competitive external review process and subsequently funded to adopt and implement a minimum of two evidence-informed interventions to offer a coordinated and comprehensive bundled intervention to improve health outcomes for Black women with HIV. One Evaluation and Technical Assistance Provider (ETAP) was also selected and funded to evaluate and disseminate implementation strategies and outcomes that can be disseminated and replicated across the RWHAP and other health care systems serving Black women with HIV [22]. A key premise of the initiative is offering Black women a package of services or evidence-informed interventions (i.e. bundled intervention) will yield better health outcomes compared to delivering an individual intervention separately [23].

This article describes the methodology employed to evaluate the adaptation, implementation, and replication of bundled interventions across the 12 sites to improve HIV health outcomes and well-being for Black women with HIV. The theoretical framework, which guides the study design and data collection methods for the adaptation of these interventions specifically for Black women with HIV is also detailed. The protocol presented is specifically designed to advance the evidence for adopting culturally responsive and relevant care into clinic and public health settings to improve the health and well-being for Black women with HIV.

## Methods

### Conceptual framework

**Fig. 1:**
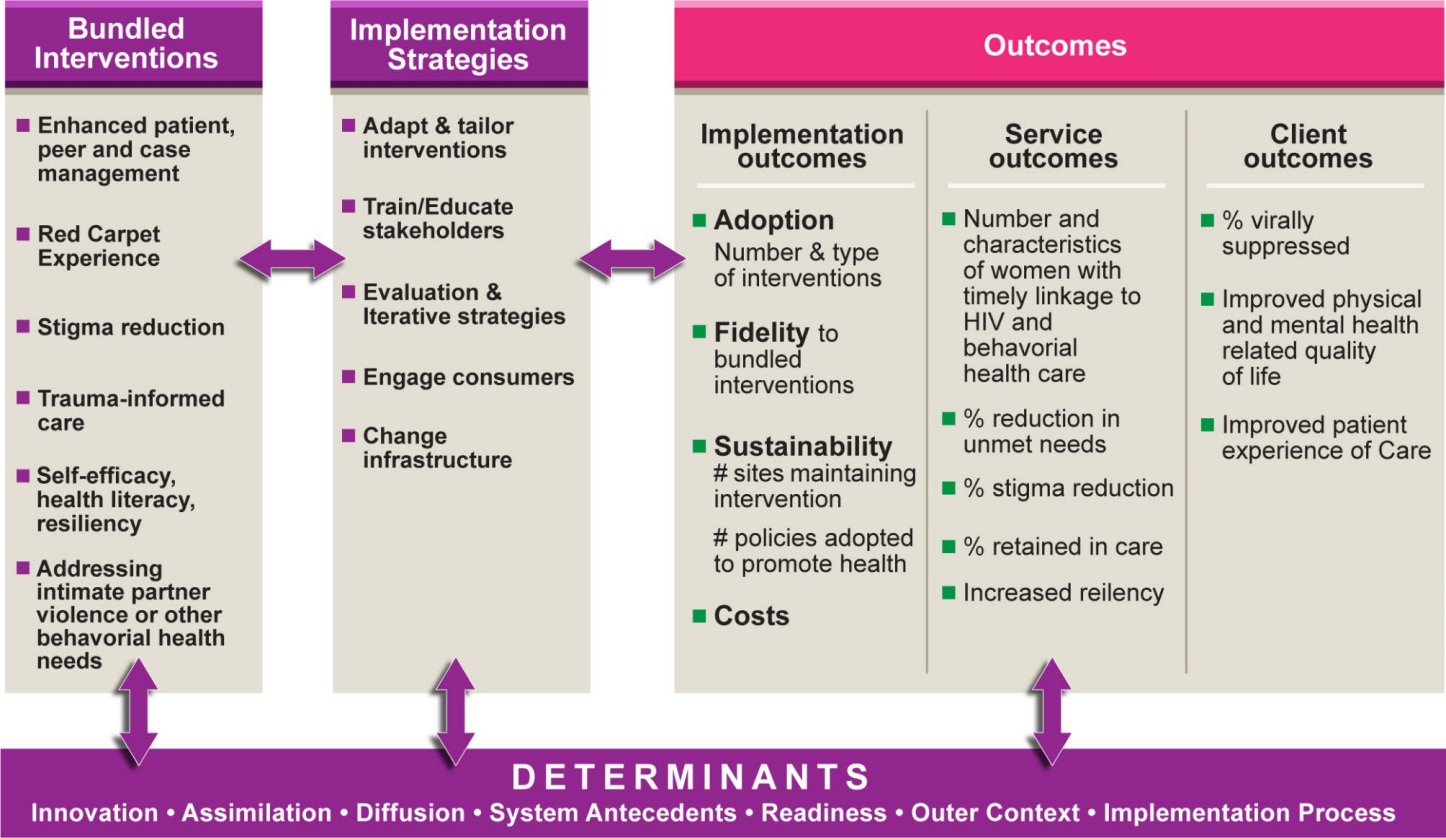
Conceptual and Evaluation Framework for the Multisite Evaluation of the Adaptation & Scaling up of Evidence- Informed Interventions for Improving Care and Treatment Coordination for Black Women with HIV

Figure 1 describes our conceptual and evaluation framework for assessing the uptake, adaptation and sustainment of the bundled intervention across the 12 sites. Data are collected to assess the effect of the interventions at the individual/participant, organizational and community levels. Our approach integrates: (1) principles of implementation science (IS) and (2) community engagement to ensure the meaningful involvement of people with HIV. In accordance with HRSA HAB’s IS work to end the HIV epidemic, the Black Women First Initiative is systematically implementing and assessing interventions, implementation strategies and impact of those interventions and strategies at the individual/participant, organizational and system levels [24].

#### Meaningful involvement of Black women with HIV

Throughout this multilevel process, we engage Black women with HIV in each step of the process to ensure the interventions, care and treatment are tailored to meet their needs. The ETAP and the 12 local sites have Advisory Councils. At the ETAP, the Advisory Council collectively has five Black cisgender and transgender women, including three women living with HIV who guide and provide feedback on the data collection and analysis and provide technical assistance to implementing the interventions. Each of the 12 sites have consumer advisory councils comprised of 4–7 Black cisgender and transgender women with HIV to provide recommendations with implementing the bundled interventions and increase outreach and retention of Black women with HIV.

Our conceptual framework for evaluating this initiative is grounded in Greenhalgh’s Conceptual Model of Diffusion of Innovations in Health Service Organizations [25] and Proctor’s model for use of implementation strategies and evaluating implementation, service and client outcomes [26–28]. Greenhalgh’s model provides the foundation for identifying and describing the key determinants that shape intervention uptake and implementation strategies within each organization and their community. The determinants include: the barriers and facilitators for the implementation process within an organization; the environmental context including the sociopolitical climate; partner networks; and leaders, structures within the organizations to support the uptake and adoption of the interventions. These determinants also influence the implementation and client outcomes on Black women’s health and well-being at each organization and across organizations.

### Description of bundled interventions

Table 1 describes the intervention domains and number of interventions adopted across the sites. Sites selected their bundled intervention from a list of HRSA HAB prescribed six domains of evidence-based/evidence-informed (EB/EI) interventions for improving health outcomes for people with HIV [22]. Each intervention domain is designed to address the individual, organization and system level factors that influence the engagement and retention in care for Black women with HIV. Each site selected the “bundled” intervention package based on the needs of their local patient populations and identification of gaps in services for Black women within their organizations or community. Below we describe briefly the six EB/EI domains.

**Table 1:** Evidence Informed interventions bundled for Improving Care and Treatment Coordination: Focus on Black Women with HIV (Black Women First Initiative) (2020–2024)

Intervention	Description	Number of sites
Enhanced Patient/Peer navigation, engagement and enhanced case management	Aims to use peer and patient navigators to provide education, coaching and social support and address the multiple needs of cisgender and transgender women of color in HIV care and treatment.	9
Red Carpet Experience	Aims to use care coordinators to reduce barriers and costs of care among “superutilizers” of health care services addressing social and medical services in HIV supporting women who are out of care and newly diagnosed	6
Stigma reduction	Aims to address stigma and the environment, use of performance arts, groups interventions and media to show HIV has a human face, engage the community, help institutions recognize stigma, educate about stigma and HIV	3
Trauma-Informed Care	Use of trauma-informed interventions, at the patient and/or organizational levels and includes training on guiding principles to a trauma-informed care approach	12
Addressing Intimate Partner Violence or other behavioral health needs	Provider/organizational level training for addressing intimate partner violence, sexual violence prevention and other behavioral health needs	6
Self-efficacy, resilience and health literacy	Group and individual interventions that increase motivation and empower women to link and engage in HIV care and improve overall physical, mental and social well-being	7

#### Enhanced peer/patient navigators and intensive case managers as part of the HIV care team to reach and engage cisgender and transgender women

Based on the findings from HRSA HAB’s Dissemination of Evidence Informed Interventions - Enhanced Patient Navigation of Women of Color [17, 29], these dedicated staff members are trained in and offer *six* structured educational sessions focused on improving HIV care, treatment and adherence, addressing the multiple social and medical needs for women as well as providing the social and emotional support and coaching for health and well-being.

#### Red carpet care experience

This intervention focuses on women who are newly diagnosed with HIV in the past 12 months or out of care for at least 6 months with the goal of linking them into care within 3 months and sustainment of care. Sites adapting this strategy to aim to support women who may be “superutilizers” of health care services and emphasize reducing the barriers and competing needs that can interfere with engagement in and adherence to HIV care and treatment. These needs include access to food, housing, employment, legal services, transportation, phone/technology, and other health services such as linkage to substance use and mental health treatment.

#### Stigma reduction

Sites are adapting and implementing community level and group interventions with peer to peer support and performance art to help women address internalized, anticipated and community stigma related to HIV.

#### Instituting trauma-informed care (TIC)

Sites are using trauma informed interventions at patient level and /or organizational level to transform the HIV health system in accordance with TIC guiding principles. At the organizational level, sites are designing and implementing formal training programs for staff and external partners. Some sites employ and integrate licensed behavioral health clinicians as part of the HIV care team to deliver evidence based therapy to enrolled women who screen for specific services. One site provides an adapted version of Trauma Recovery and Empowerment Model (TREM), an intervention for women who experienced trauma, and use substances and/or have mental health conditions. Session topics include understanding the impact of trauma on the physical body, communication, self-esteem, and self-comfort.

#### Addressing intimate partner violence or other behavioral health needs

Interventions include training staff and building organizational capacity and partner networks to screen and connect cisgender and transgender women to services such as emergency housing and behavioral health support.

#### Enhancing self-efficacy, health literacy and resiliency through the adaptation of structured group or individual evidence based interventions

Group interventions, such as Prime Time Sister Circle, designed for promoting healthy living practices for Black women in general; WILLOW [30] and TWIST [31], social support interventions for cisgender and transgender women with HIV, adapted specifically for Black women with HIV, Choosing Life: Empowerment! Action! Results! (CLEAR) [32], an individual cognitive-behavioral program for adults with or at high risk for HIV, and Heathy Relationships [33], a skills-based behavioral intervention for men and women with HIV, focusing on skills building, self-efficacy, and positive expectations about new behaviors. Other interventions such as Positive Links, a mobile technology intervention traditionally used in a predominantly men who have sex with men (MSM) population is being used to provide reminders about appointments or medication adherence and create a virtual support space for Black women with HIV [34]. With the effects of COVID-19, many of the interventions have adapted to using mobile applications or virtual learning platforms and events to engage women in services.

### Implementation strategies

To support the uptake and scale up of the bundled interventions, the Evaluation and Technical Assistance Provider (ETAP) selected five strategies from the Expert Recommendations for Implementing Change (ERIC) project [35, 36] along with assessment measures: (1) *Adapting and tailoring the bundled interventions within and across the 12 sites*; (2) *Training and educating sites and their stakeholders*; (3) *Evaluation and iterative strategies through coaching and peer to peer learning* within the organization through regular team meetings and at the multisite level. ETAP staff facilitate, coordinate, and implement monthly and semi-annual meetings to reflect and document barriers and facilitators to the implementation and replication process for the bundled interventions and strategies employed; (4) *Engaging consumers through our Advisory Council and at the sites*; and (5) *Changing infrastructure* through policies and procedures across the participating organizations and communities to promote racial and gender equity and Black women’s health and well-being through more culturally responsive care.

### Study design, data collection methods and measures

Our prospective, longitudinal study uses concurrent mixed methods (qualitative and quantitative data collection methods) to assess the implementation, service and client outcomes of the selected bundled interventions and implementation strategies to improve the HIV care continuum and well-being for Black women with HIV. Our mixed methods study documents outcomes at the client, organization, and system level. Table 2 describes our research questions in alignment with our conceptual framework.

**Table 2:** HRSA SPNS Black Women First Initiative: Multisite Evaluation Questions

*Process and implementation outcome questions*: • What are the characteristics of Black women served by bundled interventions? (Penetration) • What are the barriers and facilitators at the organizational and/or individual level to the implementation of the bundled interventions? (Adoption) • What does it cost to implement the bundled intervention? (Costs) • How is the model integrated into the mission and existing work of the site clinic/agency? (Fidelity and sustainability)	
*Service and client outcome questions*: • What is the effect of the bundled interventions on HIV care (linkage to care, retention in care, ART adherence and viral suppression) and other health outcomes (other co- morbidities such as diabetes, hypertension, and obesity, and depression, physical and mental health related quality of life)? • How do the bundled interventions address potential mediators such as stigma, discrimination, depression and unmet need for services on HIV outcomes? • What is the effect of culturally relevant and women-centered care bundled interventions on addressing stigma, discrimination at the organizational and provider level?	

Table 3 describes our data collection methods and measures across the various phases of the study. Proctor and colleagues’ model [37, 38] guides and defines the evaluation outcomes for the initiative.

Qualitative data are collected systematically primarily through a series of *annual site visits and questions guides* and *a standardized monthly call form* with participating site staff that capture barriers and facilitators to the implementation process as well as the key determinants impacting the intervention uptake and implementation strategies. Quantitative data collection for the implementation, service and client outcomes is conducted through a pre-post prospective study to examine the impact on Black women’s health and well-being. Three data collection tools: *client interviews, medical chart review, and an intervention encounter form* gather service and client outcome data. Interview and chart data are collected with women enrolled in the study at baseline (prior to receipt of the bundled interventions) and at 6 and 12 month follow-ups. Intervention staff complete daily encounter forms to document the frequency, mode of delivery, content, and duration of intervention services provided to participants. These tools were developed and piloted with participating sites prior to the launch of the study.

#### Eligibility criteria

Individuals are referred, recruited and screened for enrollment in the intervention. Eligibility for participation includes: 18 years or older, identify as Black or African American, identify as cisgender or transgender female and have a diagnosis of HIV. Pregnant women are also included in our study. Across the 12 sites and depending on the selection of the bundled intervention services, some sites have further inclusion criteria such as: women who are newly diagnosed with HIV (defined as within 12 months of enrollment date) or been out of care for six months or longer prior to enrollment, or not virally suppressed.

*Implementation outcomes* include: socio-demographic characteristics of and risk factors for Black women served by the bundled interventions *(penetration*; e.g., history of trauma, incarceration, housing and employment stability, food security); the *adoption* of interventions across the sites by number and type of intervention, the staffing and partner model within the agency and across the community; the *fidelity* to the components of the bundled interventions; the *costs* of the intervention; and the *sustainability* of the intervention in the organization (changes in organization culture and policies) and community.

*Primary service and client outcomes* are related to the HIV care continuum in alignment with the HRSA core performance measures: *Linkage to care within 30 days of enrollment; retention in care* defined as two appointments at least 90 days apart in the 12 month period post enrollment; and *prescribed antiretroviral treatment* in the 12 months post enrollment period; viral suppression < 200 copies/ml in 12 months post enrollment; and *durable viral suppression* as at least 2 consecutive viral load tests < 200 copies/ml in the study period. Secondary clinical care outcomes include a self-report three-item scale on adherence to HIV medications and access and engagement in behavioral services and preventive health screening measures. All these data are gathered via medical chart review.

*Other measures*: In addition to HIV health outcomes, other self-report service and client outcomes include: unmet need for services, social support networks, experience with stigma and discrimination and resilience, health literacy, physical and mental health related quality of life, and perceived patient experience of care. *Unmet needs for services* consists of 14 social and behavioral health service needs including housing, transportation, employment, legal services and child care. Clients are asked if they needed the service (yes/no) and if they were able to get it (yes/no) in the past 6 months. The 12-item *Multidimensional Scale of Social Support* [39] measures the number and type of social support networks. The *HIV Stigma Scale* [40] is a 13-item scale that assesses both internal (shame, negative self-image) and perceived external stigma from family, friends, employers, and providers and a seven item *discrimination* scale to assess experiences over time with poor treatment due to racial and ethnic identity. A four-item scale assesses client *health literacy* [41] comprehension of medical information. *Resiliency and coping* [42] is measured using a three-item scale to assess the effectiveness of the interventions on improving women’s ability to address challenges and problem solve life situations. Finally, we measure *quality of life* using two validated scales measuring physical and mental health function and life satisfaction [43, 44].

### Process and outcomes data collection across the organizational and system levels

In addition to the longitudinal study at the client level, qualitative and quantitative data collection methods are used to capture intervention uptake, adaptation and strategies utilized at the organizational and community levels. Qualitative data were collected during the *pre-implementation phase (Year 1)* using a standardized initial needs assessment tool and key informant question guides with site teams. The assessment tool and question guide were developed based on Greenhalgh’s determinants and gathered data on the types of interventions selected, the sites’ readiness and absorptive capacity, their resources and systems, the diffusion and dissemination process within the site and their community and the outer context including policies, partner networks, and socio-political and economic climate that could impact intervention uptake and scale-up. The data analyses are currently underway.

To complement qualitative data collection at the organizational level, a longitudinal study is being conducted using *the Organization Readiness for Implementation Change (ORIC) Survey* [45, 46]. This survey was delivered to site and partner staff at baseline (prior to the launch of the interventions) and then post 6 and 12 months. The ORIC survey consists of 12 items that assess sites’ system antecedents and readiness to adopt and implement the bundled interventions. The items measure both change efficacy and commitment [45, 46] at an organizational level. The aim of this sub study coupled with qualitative data may provide valuable lessons learned and practice recommendations for other clinical and community organizations working to improve Black women’s health outcomes. A copy of our survey is provided in the Supplementary material 1.

During the implementation phase (Years 2–3*)*, we document the implementation strategies, adaptations to the interventions and activities through qualitative and quantitative methods. Qualitative data are collected using a standardized site monthly call form and annual site visit guide with site staff based on the key determinants in Greenhalgh’s model. Information is collected on organizational factors (i.e., leadership, dedicated resources, communication channels, referral and quality improvement systems, and partner networks); and barriers and facilitators to intervention uptake, such as new funding, training and technical assistance activities and staff turnover. Sites also reported on changes in polices to promote racial and gender equity as part of the initiative. In addition, key questions based on FRAME-IS [47] are added to monthly call forms to capture modifications made to intervention and implementation strategies. These elements include: description of modification by content, staff, timing and mode of delivery, the decision makers involved in modifications, and the goal and rationale for the modification [47]. In the final year of implementation (Year 3) key informant interviews are planned with site and implementing partner staff to gain information on the experience with the bundled interventions, impact on organizational and community policies and structures and perceptions on adapting sustaining interventions for Black cisgender and transgender women with HIV. Photovoice projects are also planned with selected sites and participating clients to capture participant experience with the bundled interventions. A copy of our pre-implementation and implementation guides are provided in Supplemental Material 2.

Concurrently during the implementation phase, site staff collect data via daily intervention encounter forms to document and measure *fidelity* to the bundled intervention and activities, frequency, mode of delivery and duration. The encounter forms, developed with site staff and ETAP Advisory Council members, describe the services, staff delivering the services and activities related to medical, behavioral health, food, housing and employment, education and emotional support. Combined with the qualitative data, this information identifies the key elements for culturally relevant care for Black women with HIV.

As part of implementation outcomes for the organizational level, the initiative is collecting data from the sites on factors associated with *replication* and *sustainability and cost.* To assess program sustainability, sites will be trained to use the Program Sustainability Assessment Tool (PSAT) [48] to identify and plan for sustained uptake of the bundled interventions into their agency and to scale up to other populations in their community. The ETAP will further assess success with sustainability by conducting a survey 6-months post conclusion of site funding to determine continued implementation of bundled interventions. Finally, a cost analysis is planned to provide recommendations to other HIV and public health entities on replicating the bundled interventions to reach Black women with HIV. Our cost analysis is based on a *provider perspective* with data elements collected from the health system or care provider. A standardized cost tracking form is used to collect data on different components of the intervention, including the costs of personnel, fringe benefits, supervision and training, clinical support services, materials and support for clients and staff and other direct and indirect costs. Our analyses will include the cost per person recruited into care and cost per person retained in care and virally suppressed.

All study materials were approved by the Institutional Review Boards (IRB) of the ETAP (University of Massachusetts Lowell and Boston University Charles River and Medical Campus). The 12 participating demonstration sites also received IRB approval for the longitudinal study on implementation and service outcomes at the client level.

**Table 3:** Key measures and methods for Evaluating the Implementation and Replication of Bundled Interventions to Improve Care and Treatment Coordination for Black women with HIV

Process Evaluation			
Phase	Determinants/Elements	Measures	Data collection method
Pre-implementation	*Complexity and Relative advantage of intervention* *System readiness* *System antecedents* *Diffusion* *Adoption/Assimilation* *Outer context*	• Innovation- bundled interventions and key characteristics of women and organizations • Organizational factors: Readiness and absorptive capacity; infrastructure, leadership, organizational assets, culture, and climate, supervision and referral systems; partner networks; quality improvement and data systems for feedback; • Structural factors: poverty, racism, intimate partner violence; services and policies	• Key informant interviews Year 1 35–40 (3/ per site) • TA Needs Assessment • Organizational Readiness for Implementation Change (ORIC)
Implementation	*Implementation process* *Adoption/Assimilation* *Diffusion and Dissemination* *Outer Context*	• Leadership, dedicated resources, internal and external communications and collaboration and feedback mechanisms on progress. • Facilitators and barriers to implementation (staff turnover, funding, training/TA) • Adaptations to intervention bundles • Documentation of implementation strategies • Structural factors	• Annual Key informant interviews (Yr. 3) site staff • Staff intervention encounter form (ongoing-Years 1–3) • Monthly call forms in REDCap (ongoing Years 1–3)
**Outcome Evaluation**			
*Post* *implementation*	*Implementation* *Outcomes*	• *Penetration*: Socio-demographics and social risks (food security, housing and employment, health literacy,) of Black women with HIV and types of organizations • *Adoption*: Number and type of interventions for Black women with HIV adapted across the 12 sites (quantitative); • *Fidelity* to the selected intervention (e.g., Peers/Patient Navigators trained, supervision and referral systems (qualitative); dose, duration and types of activities (quantitative) • *Sustainability*: # of sites sustaining interventions; # of policies adopted to promote health for Black women with HIV • *Cost*: Fixed vs. variable: Personnel, Materials, Staff client transportation, Agency indirect rates; start up and implementation from Provider Perspective	• Client baseline survey • Enrollment reports • Monthly call forms and Site visits • Staff Intervention encounter form • Program Sustainability Tool and Survey (PSAT) • Cost analysis worksheet-Annual Fiscal and administrative records
		• Client /Community case studies of experience with intervention • Barriers and facilitators for seeking and maintaining intervention	Community Participatory Research In-depth client interviews (Photovoice) Focused studies
	*Service Outcomes*	*HIV care and behavioral health care*: Timely linkage and retention in care; Unmet needs for services, barriers to care (personal beliefs, organizational, structural) Alcohol and substance use, mental health, HIV stigma, gender-race discrimination, Violence and trauma, Social support and networks, Resilience; health literacy; perceived quality of women-centered care	Medical Chart review, Client survey Baseline, 6 and 12 months
	*Client outcomes*	*HIV care outcomes*: ART adherence, viral suppression *Other health outcomes*: co-morbidities (diabetes, depression) *Patient experience with care, quality of life (physical and mental health functioning, life satisfaction;*	Medical Chart review, Client survey Baseline, 6 and 12 months

## Discussion

In the United States, pervasive racial inequities in HIV treatment exist. Black women are among the most disproportionately impacted by HIV and underserved populations that experience treatment inequities across the care continuum. Simultaneously, there is great variation within the Black population and to date few efforts have sought to tailor interventions to meet the needs of diverse Black women. One of the key indicators for the goal of reducing HIV health disparities in the National HIV/AIDS Strategy (2022–2025) is to increase the viral suppression rates among Black women diagnosed with HIV from 59 to 95% [1]. To meet this goal, the health service delivery system needs to transform and address the multiple challenges and historical trauma Black women face due to racism, sexism, classism, lack of social capital, compounded by HIV stigma and discrimination. Evidence based/evidence informed interventions aimed at improving the HIV care and treatment for people with HIV have largely focused on men with more recent studies testing EB/EI interventions for transgender women. Implementation gaps persist for Black cis and transgender women across the HIV care continuum. This initiative will contribute to the evidence for key strategies to provide culturally relevant care for Black women with HIV across the adult lifespan that improves their HIV health outcomes and well-being. The Improving Care and Treatment for Black Women with HIV (Black Women First) Initiative aims to share the findings on the adaptations of these interventions and strategies with other Ryan White HIV/AIDS Programs (RWHAP) and health care organizations so they can more effectively center the health care priorities and better reach and serve Black women.

**Table 4:** Participating study sites for the HRSA/SPNS Improving Care and Treatment Coordination: Focus on Black women with HIV

Site Name	IRB Name	IRB Approval Number
Positive Impact Health Center	WIRB-Copernicus Group (WCG) IRB	1306382
Grady Health Systems	Research Oversight Committee IRB	000-2140
Quality comprehensive Health center	Sterling IRB	8879-LWigfall
Access Matters	Heartland IRB	052821-326
AIDS Foundation Chicago	Solutions IRB	2021/03/7
AIDS Care Group	WIRB-Copernicus Group (WCG) IRB	1304505
Volunteers of America Southeast Louisiana	Solutions IRB	2020/10/3
Institute of Women and Ethnic Studies (IWES)	IWES IRB	A00015805
Abounding Prosperity	Jackson State University IRB	0067-21.22
Alliance for Positive Change	Brany IRB	RH2015-101
City of Philadelphia (CoP)	CoP Department of Public Health IRB	2021-20
University of California San Francisco (UCSF)	UCSF Health IRB	21-33408

Moreover, the study protocol developed for this initiative may advance the implementation science field by furthering what is known about the ways in which bundled interventions can address barriers to care and facilitate the uptake of organizational practices to improve health care for Black women. There is limited evidence of the effectiveness of bundled interventions in HIV services and the specific mechanisms associated with bundles that improve care. Involving Black women in all aspects of this initiative may contribute to knowledge production that supports the design and delivery of better health and social care, while also informing the development of policies to address racial and gender inequities.

## Electronic supplementary material

Below is the link to the electronic supplementary material.

Supplementary Material 1: Organizational readiness survey

Supplementary Material 2: Pre-implementation & implementation study guides

## Data Availability

There are current datasets generated and/or analyzed during for this protocol. All data collection materials are available from the corresponding author on reasonable request.
