# Supplementary material for: The Black women first initiative: using implementation science to examine bundled interventions to improve care and treatment coordination for Black women with HIV
Source: BMC Health Serv Res. 2023 May 26;23:551. doi: 10.1186/s12913-023-09446-z (PMC10214367; doi:10.1186/s12913-023-09446-z)
Supplement: Supplementary file 1 — Supplementary Material 1: Organizational readiness survey [file 12913_2023_9446_MOESM1_ESM.pdf]

Organizational Readiness Baseline Survey

Dear [name],

Thank you for agreeing to participate in this brief assessment to understand your organization's capacity and readiness for implementing the HRSA SPNS Project, [intervention], to improve care and treatment coordination for Black women with HIV. You are being asked to complete this survey because a member of your organization or a partner agency identified you as playing a key role in this intervention. The purpose of the survey is to gather information about factors that may affect an organization's success with implementing the intervention. In addition to this initial survey, we will reach out to you to complete a follow up survey at two additional times in the next 12 months.

We would like to remind you quickly of key points before starting the survey:

Organizational readiness for implementation, defined as "tangible and immediate indicators of organizational commitment to its decision to implement an intervention" is an integral precursor to successful implementation. The survey consists of questions about personnel demographics and organization characteristics and questions about your organizational readiness to implement the bundled intervention. Readiness questions are adapted from the Organizational Readiness for Implementing Change (ORIC). This 12-item instrument is used to determine how well employees at an organization feel they can implement the change in processes required by a proposed intervention. Each item includes a Likert scale from 1 (Disagree) to 5 (Agree).To gain a broader perspective, the survey is administered to multiple individuals within the organization or partners that have different roles relevant to the intervention or decision making around the intervention. General instructions:

The survey will take approximately 15-20 minutes to complete. Your participation is voluntary, and you can withdraw from the study during the survey round without comment or penalty by closing the survey link. By clicking (yes, I consent) on the first page, you will indicate that you have fully read and understood the complete information document provided to you earlier regarding this study with the participant invitation/recruitment email. Your responses to the survey are automatically saved as you go through the questions, and at any moment you can close the survey link and continue it later by using your same personal link. All data collected and processed will be kept anonymous, confidential, and stored on a password-protected database at the University of Massachusetts Lowell.

Email address (hidden field)

____

Name (hidden field)

____

Organizational affiliation (hidden field)

Name of bundled intervention (hidden field)

Today's date: ________________________________

SECTION I: PERSONNEL DEMOGRAPHICS

1. How old are you? ________________________________ (years)

2. What is your gender identity?

- ◯ Man
- ◯ Transgender man / Trans man
- ◯ Woman
- ◯ Transgender woman / Trans woman
- ◯ Gender variant/ Non-conforming
- ◯ Additional identity (specify below)
- ◯ Declined

If additional identity, please specify: ________________________________

3. Are you of Hispanic, Latino/a, or Spanish origin? (A person of Cuban, Dominican, Mexican, Puerto Rican, South or Central American, or other Spanish culture or origin, regardless of race)

- ◯ Yes
- ◯ No
- ◯ Declined

4. What race do you consider yourself? (Please answer yes or no for each of the following. You may say yes to more than one.)

☐ Black or African American
☐ Asian
☐ Native Hawaiian or other Pacific Islander Alaska Native
☐ White
☐ American Indian
☐ Other (specify below)
☐ Declined

If other, please specify: ______________________________

5. What is the highest level of education that you've completed? (One response only)

◯ No formal education
◯ Middle school (Junior High School) or less
◯ Less than High School
◯ High School Diploma or GED Received
◯ Some junior college (2-year college)
◯ Junior (2-year) college
◯ Technical/trade/vocational school
◯ Some college (4 year college or university)
◯ College graduate (4-year college or university)
◯ Post-college/graduate
◯ Don't know
◯ Declined

6. Which of the following best describes your role?

☐ Community health worker
☐ Peer navigator/advocate
☐ Case manager
☐ Social worker
☐ Mental health provider (psychiatrist, LMHC, LICSW, LCSW, etc)
☐ Primary care provider
☐ Administrator
☐ Evaluator/Quality Improvement Manager
☐ Other (please describe)

If other, please describe: ______________________________

7. How long have you worked in this role? ______________________________

8. How long have you worked with your current organization? ______________________________

SECTION II: ORGANIZATIONAL CHARACTERISTICS

9. Are you affiliated with [site] or are you a subcontractor?

◯ [site]
◯ Subcontractor to [site]

10. Has your organization participated in a previous HRSA/SPNS project

◯ Yes
◯ No
◯ Don't know

---

11. Which of the following best describes your organization?

◯ Health center/hospital
◯ Community based organization
◯ AIDS service organization
◯ Health department
◯ Other (specify below)

If other, please describe:

____

---

12. Annual number of patients/clients with HIV seen at your organization

◯ 0 - 500
◯ 501 - 1,000
◯ 1,001 - 1,500
◯ 1,501 - 2,000
◯ 2,001+

---

13. How would you best describe the geographic location of your organization?

◯ Urban setting
◯ Suburban setting
◯ Rural setting

---

14. Which of the following best describes your organization's service area?

◯ 1 county
◯ 2 - 3 counties
◯ 3 - 4 counties
◯ 5 - 6 counties
◯ 7 - 8 counties
◯ 9+ counties

---

15. Other federal funding available

☐ Ryan White Part A
☐ Ryan White Part B
☐ Ryan White Part C
☐ HRSA Health Center (330 funds)
☐ CDC HIV prevention or Ending the HIV Epidemic (EtHE)
☐ HOPWA/HUD
☐ CARES Act (federal COVID relief)
☐ Other (specify below)

If other, please describe:

____

16. What are the bundled interventions that are being carried out at your organization for this HRSA SPNS project, [intervention]? (Select all that apply)

☐ Enhanced patient navigation, enhanced case management, peer engagement
☐ Red Carpet care experience
☐ Stigma reduction interventions
☐ Trauma-informed Interventions (organizational capacity building)
☐ Intimate Partner Violence (organizational training, screening & assessments)
☐ Self-efficacy, health literacy & resilience interventions
☐ Other (specify below)

If other, please describe:

____

17. Which of the following best describes your priority population for [intervention]? (Select all that apply)

☐ Cis women
☐ Trans women
☐ Women >55 years
☐ Women < 26 years
☐ Women with multiple co-morbidities
☐ Women who are experiencing homelessness/unstably housed
☐ Women experiencing violence (Intimate Partner Violence, Domestic Violence, etc.)
☐ Women across the diaspora (specify any immigrant populations below)
☐ Other (describe below)

Please specify immigrant populations:

____

If other, please describe:

____

This set of questions asks about the organizational readiness for delivering bundled interventions in your organization.

Please read each statement and indicate the response that best reflects your organization's readiness to implement [intervention].

	Disagree	Somewhat Disagree	Neither Agree nor Disagree	Somewhat Agree	Agree
1. People who work here feel confident that the organization can get people invested in implementing a bundled intervention to improving care and treatment coordination for Black women with HIV.	○	○	○	○	○

2. People who work here are committed to implementing a bundled intervention to improving care and treatment coordination for Black women with HIV. ○ ○ ○ ○ ○

3. People who work here feel confident that they can keep track of progress in implementing a bundled intervention to improving care and treatment coordination for Black women with HIV. ○ ○ ○ ○ ○

4. People who work here will do whatever it takes to implement a bundled intervention to improving care and treatment coordination for Black women with HIV. ○ ○ ○ ○ ○

5. People who work here feel confident that the organization can support people as they adjust to implementing a bundled intervention to improving care and treatment coordination for Black women with HIV. ○ ○ ○ ○ ○

6. People who work here want to Implement a bundled intervention to improving care and treatment coordination for Black women with HIV. ○ ○ ○ ○ ○

7. People who work here feel confident that they can keep the momentum going in implementing a bundled intervention to improving care and treatment coordination for Black women with HIV. ○ ○ ○ ○ ○

8. People who work here feel confident that they can handle the challenges that might arise in implementing a bundled intervention to improving care and treatment coordination for Black women with HIV. ○ ○ ○ ○ ○

7. People who work here are determined to implement a bundled intervention to improving care and treatment coordination for Black women with HIV.	○	○	○	○	○
10. People who work here feel confident that they can coordinate tasks so that implementing a bundled intervention to improving care and treatment coordination for Black women with HIV goes smoothly.	○	○	○	○	○
11. People who work here are motivated to implement a bundled intervention to improving care and treatment coordination for Black women with HIV.	○	○	○	○	○
12. People who work here feel confident that they can manage the politics of implementing a bundled intervention to improving care and treatment coordination for Black women with HIV.	○	○	○	○	○

What are the 2 main challenges that you have encountered when implementing [intervention] for Black women with HIV? (These challenges could be related to your organization or from the larger community)

____

What has helped you with implementing [intervention] for Black women with HIV?

____
