# Supplementary material for: The Black women first initiative: using implementation science to examine bundled interventions to improve care and treatment coordination for Black women with HIV
Source: BMC Health Serv Res. 2023 May 26;23:551. doi: 10.1186/s12913-023-09446-z (PMC10214367; doi:10.1186/s12913-023-09446-z)
Supplement: Supplementary file 2 — Supplementary Material 2: Pre-implementation & implementation study guides [file 12913_2023_9446_MOESM2_ESM.pdf]

Black Women First ETAP Site Assessment Tool
November Monthly Call Form
Pre-implementation Study Question guide

Site:
Date:
Participants from Site:
Participants from ETAP:

As part of the multisite evaluation we are interested in talking with leadership and staff across all 12 sites who are involved in the implementation and evaluation of the bundled interventions for Black Women with HIV. Our goal is to learn more about your overall approach and initial plans for implementation. We also want to learn more about resources and assets you are building on as well as anticipated challenges for implementing the bundle interventions for Black women with HIV. We are also very interested in hearing about your strengths and experiences as well as areas in which you would like additional support. This information will inform peer learning opportunities across sites and technical assistance plans.

A. Innovation
   1) In your application you discussed a number of health care inequities experienced by Black women living with HIV. Can you talk about the components of your proposed bundle and how the bundle will advance service delivery/access reducing health care inequities experienced by Black woman living with HIV in your area?
      a. How is this approach different from your previous attempts?
      b. What you think about the root causes of health care inequities experienced by Black women living with HIV how will this intervention begin to address them? (Probe: Are there factors that affect cisgender and transgender women?)
      c. For your perspective what is innovative about what you are proposing to do and how you are proposing to do it?

   2) Why did you select the specific interventions for this SPNS initiative to improve care and treatment for Black women with HIV?
      a. What was the role of organizational stakeholders (staff, clients, community partners) in your selection decision? Can you talk specifically about how Black women living with HIV informed selection decisions?
      b. How does the intervention align with your agency's mission and values?
      c. If you are able to achieve your intended goals with this intervention, how will the agency and broader community benefit? How will the experiences of Black women living with HIV in your area be different? How might experiences vary for cisgender and transgender women?
      d. What resources and assets will you leverage to implement the bundled intervention? Consider those both internal and external to your organization (i.e.: staff, partners, technical experts, financial resources) Who are you existing partners for this initiative? Who are you new partners for this initiative? What technical support do you anticipate you will need to adapt the interventions in your agency? In your community?

B. Adoption
   a. How are you planning to deliver the various interventions as a bundled intervention? By whom are they being delivered?
   b. When do you anticipate your agency will be ready to implement the intervention (s)?
   c. What factors do you foresee will be a barrier to implementing the intervention in your agency? In your community?
   d. Who are the key staff and stakeholders that will support uptake of the intervention in your agency? How are you engaging stakeholders such as clients and or community partners in the implementation process? Specifically, how will Black living with HIV be involved in decision-making related to implementation and adaptation?

C. Fidelity to the intervention
   a. Sometime implementation teams use manuals, protocols or guides when they are putting new services in place. Do you have a road map you are following to implement your bundle? (Probe: Are there particular elements of the bundle that have a blueprint or guide? are there any elements that come with TA?) Do you have technical expertise on site or with partner orgs related to elements of your bundle? Would you be open to sharing documents you are using to guide your work? What types of support or technical assistance might you needed related to developing or adapting implementation materials?
   b. To what degree is the intervention being implemented as originally designed? How are you planning to document changes that are made to the intervention along the way?
      a)How are you adapting elements of your bundle intervention?

D. Implementation process
   a. How is this intervention being integrated into the services provided at your site?
      a) How is leadership supporting the integration of this intervention?
   b. Have you designed any materials (patient or community) to support the implementation of this intervention? Please attach them to this call form.
   c. What is the referral and recruitment process for women into the interventions?
   d. What are the feedback mechanisms for progress with the intervention?
   e. What policies and programs are in place to support the intervention at your agency? What policies need to be developed? Who will be involved?
   f. What other funding will you leverage to implement these bundled interventions?

E. Outer context
   a. Communities are complex lots of different actors and agencies, norms and dynamics. We want to understand your work and what you have proposed in the broader community context. Can you talk a little bit about the local landscape… What are the community conditions Black woman living with HIV are experiencing? What does the local service landscape look like from their perspective? How does your program fit in the context of the broader community and local service landscape? How do you think your proposed intervention will impact the broader service landscape?

b. How has the COVID-19 pandemic impacted your organization? How has the COVID-19 pandemic affected the clients you serve?
   a) Are you conducting services virtually? Please describe.
   b)How has the movement for Black lives and efforts to tackle structural racism impacted your work? (probe: How has structural racism impact your ability and capacity to access resources to serve the community? Engage in advocacy?)
   c)What efforts has your organization made to focus more on the role of structural racism?
c. In light of the COVID-19 pandemic how will the intervention (s) be adapted?
d. How is trauma considered in your work? If you engage in trauma-informed care, how do you define trauma informed care?
e. How does your SPNS intervention fit into the jurisdictional plan to End the Epidemic (EtHE)? How will EtHE plan affect the implementation of this intervention?

F. **Staffing updates** (including new contractors, new staff members, major administrative changes in the organization, integration of intervention team members into the clinical team)

G. Can you talk a little about the staff plan associated with your bundled intervention? To what extent were positions prescribed by guidelines associated with bundle elements? How well aligned with your staffing were positions required by implementation guides associated with elements of your bundle?
   a. What is the status of your hiring process for SPNS intervention staff?
      1. What is the process and timeline to fill the vacancy?
      2. What is the process to onboard and train new staff on the bundled interventions?
   b. Have you experienced any staff turnover in the past 6 months?
      1. If yes, why did the staff person leave?
   c. Has your staff participated in any trainings (offered either by your clinic/organization, a community partner, national partners, etc.)?
      1. Training topic:
      2. Training modality (in person, online):
      3. Notes:
   d. In the past month, how often did intervention staff receive administrative supervision? Clinical supervision?

H. **Evaluation updates**
   a. How is your site identifying eligible clients to be served by this project?
      a)Describe the workflow and how will it be decided who gets what?

   b. How many participants do you plan to enroll in the program and evaluate outcomes
      1. What screening tools will you use to determine eligibility?
      2. What barriers or challenges do you anticipate to implementing the evaluation activities?

I. **Technical assistance needed**

a. Of the bundled interventions, which ones are you already trained on?
    a) Are all staff members who will implement the bundled intervention trained?
    b) If already trained, please describe staff's experience implementing the bundled interventions.
    c) Is any technical assistance or booster training needed to support your implementation of the bundled intervention?
b. Of these bundled interventions, which ones do you need training on?
    a) What is your plan to secure training on these interventions?
    b) How many staff need training on the interventions?
c. What is your team's plan to offer professional development to staff implement the bundled intervention?
d. What other training topics are of interest to your team?
    a) We could potentially add a laundry list for sites to choose from in this area?
        - Stigma
        - Resiliency
        - Supervision
        - Meaningful Involvement of People with HIV
        - Motivational Interviewing
        - Mental health first aid
        - Trauma-informed Care
        - Cultural humility
        - Counseling
        - Other (please describe)
e. What would you like to learn from other sites? What experiences and skills do you have that you would be willing to share with other sites?
f. We know that in person training is preferred, but how open is your team to receiving training virtually?
g. What equipment does staff have to ensure full engagement in virtual training?
    a) Computer
    b) Functional webcam
    c) Audio
    d) Headset

Black Women First ETAP
Ongoing Monthly Site Call Question guide
HRSA-Sites

Organization :
Today's Date :

Program update: Please provide an update related to program activities and implementation since the last monitoring call.

- How are the various interventions being delivered as a bundled intervention?
- How is this intervention being integrated into the services provided at your site?
  - How is leadership supporting the integration of this intervention?
- Are you conducting services virtually?
  - If yes, please describe which activities are being implemented virtually at your organization? Which activities related to the bundled intervention?
- Have you made any modifications or adaptations to your intervention since the last call?
  - If yes, please describe the context for the modification or adaptation:
    - What is modified or adapted?
    - What was the goal of the modification or adaptation?
    - Why is the modification taking place?
    - Who participated in the decision to modify or adapt?
    - When will the modification or adaptation be put into place?
    - Other comments:

- What has helped you implement the bundled intervention?
- What challenges are you experiencing with implementing the intervention?
- What strategies are you using to address any challenges to the implementation?
- How has the COVID-19 pandemic impacted your organization? How are the new variants affecting your organization (if at all)?
- How has monkeypox impacted your clients, organization and/or partners? How has monkeypox impacted this SPNS initiative?
- Who are your new partners?
- How have you worked with your community advisory board (CAB) this past month?
- How is your organization and your partners supporting Black women with managing life in this time of the COVID-19 pandemic? *(e.g. helping with vaccination efforts, testing, education, providing food and shelter support, etc…)*
- Have there been any major changes within your agency/clinic or community that may influence the implementation of your intervention(s)? *(e.g. changes in funding, community events or news stories, opening new clinic locations, local or national policies that impact your work with your patients)*
- Have you designed any materials (patient or community) to support the implementation of these interventions?
- (First month) How do you define racial equity within your team and organization?

- (Subsequent months) Has your definition or understanding of racial equity changed within your team or organization?
- How do you see racial equity appearing in your work currently? *(e.g. policies, interactions, trainings, etc)*
- How are you implementing ideas from your racial equity action plan? What is going well? What is not going well? What would you like support around?

- **Staffing & Training updates** (including new contractors, new staff members, major administrative changes in the organization, integration of intervention team members into the clinical team)
  - Have you experienced any staff turnover?
    - If yes, share the name and position of the individual(s) who left.
    - Why did the staff person(s) leave? (Please provide as much information as confidentiality will allow —i.e. promotion to new position in the agency, moved out of the area, decided to pursue further education, obtained a job in another agency but related field, career change, family responsibilities, medical leave...)
    - What is the process and timeline to fill the vacancy?
    - What is the process to onboard and train new staff on the bundled interventions?
  - Have your staff participated in any trainings (offered either by your clinic/organization, a community partner, national partners, etc.)? *(If your staff have participated in multiple trainings, please provide details for each training separately below.)*
    - Name/topic of first training:
    - Number of training hours completed:
    - Training modality (in person, online):
    - Staff member(s) who participated in this training:
    - Notes:
    - *Spaces for second and third training listings in REDCap
  - In the past month, how often did intervention staff receive administrative supervision?
  - In the past month, how often did intervention staff receive clinical supervision?

- **Evaluation updates**
  - How is your site identifying eligible clients to be served by this project?

  - How many clients received SPNS services in the past month? ___
    - How many were new to the programs? ____
    - How many total enrolled in the evaluation study?

  - What recruiting strategies are you using to identifying clients for the program?

  - What barriers or challenges do you have implementing the evaluation activities?

- **Dissemination updates**
  - How are you sharing information about the SPNS intervention to the wider community? This could be with the Ryan White Planning Councils, Ending the Epidemic committees, other entities focused on Black women's health.
    - Name the event, the activity and staff or partners involved?

- **Technical assistance needed**
- Did you receive any TA or training from the ETAP and/or bundled intervention technical experts over the past month?
  - If so, please describe.
- Do you have any feedback on the TA or Training provided?
- What additional TA or training do you need?
- What topics would you like to discuss with other SPNS sites?

- **Scheduling**
  - Confirm next call time/date
  - If relevant, confirm upcoming site visit or training dates

- **Q&A**
